# Long-term immune recovery under continuous antiretroviral therapy (ART) among ART-naive people living with HIV in two cohorts in Germany

**DOI:** 10.1007/s15010-026-02740-y

**Published:** 2026-02-26

**Authors:** Uwe Koppe, Kirsten Pörtner, Christian Kollan, Annemarie Pantke, Andrea Sailer, Kerstin Dehmel, Stefan Esser, Björn-Erik Ole Jensen, Jürgen Kurt Rockstroh, Guido Schäfer, Dirk Schürmann, Matthias Stoll, Jan-Christian Wasmuth, Tobias Kurth, Barbara Gunsenheimer-Bartmeyer

**Affiliations:** 1 Department of Infectious Disease Epidemiology, Robert Koch Institute, Seestr. 10, 13353 Berlin, Germany; 2 Department of Dermatology, University Hospital Essen, University Duisburg-Essen, Essen, Germany; 3 Department of Gastroenterology, Hepatology and Infectious Diseases, Medical Faculty and University Hospital Düsseldorf, Heinrich Heine University Düsseldorf, Düsseldorf, Germany; 4 Department of Medicine I, University Hospital Bonn, Bonn, Germany; 5 Infektionsmedizinisches Centrum Hamburg, MVZ ICH Grindel, Hamburg, Germany; 6 Department of Infectious Diseases and Pulmonary Medicine, Charité - Universitätsmedizin Berlin, Berlin, Germany; 7 Department of Rheumatology and Immunology, Hannover Medical School, Hannover, Germany; 8 Institute of Public Health, Charité - Universitätsmedizin Berlin, Berlin, Germany

**Keywords:** HIV, CD4, CD4/CD8 ratio, Immune recovery, Long-term treatment, Antiretroviral

## Abstract

**Objectives:**

Immune recovery among people living with HIV (PWHIV) receiving antiretroviral therapy (ART) is determined analysing CD4 cell counts and the CD4/CD8 ratio. Recovery to ≥ 800 CD4 cells/µl and CD4/CD8 ratio ≥ 1 was associated with favourable outcomes. We investigated immune recovery over 10 years among ART-naive PWHIV on ART.

**Methods:**

Data were obtained from two German HIV cohorts, the HIV-1 Seroconverter study and the ClinSurv-HIV study, between 2003 and 2018. We included ART-naïve PWHIV starting with continuous ART and analysed CD4 cell counts and the CD4/CD8 ratio. The time to reaching immune thresholds was investigated using Kaplan–Meier analyses with inverse probability censoring weights.

**Results:**

Overall, 8,927 participants were included. At baseline, PWHIV had a median CD4 cell count of 257 (interquartile range [IQR] 124–393) cells/µl and CD4/CD8 ratio of 0.26 (IQR 0.14–0.43). After ten years, median CD4 counts increased to 630 (IQR 474–811) cells/µl and CD4/CD8 ratio to 0.84 (IQR 0.61–1.11). PWHIV with higher baseline CD4 values and without viral failure had higher median CD4 counts and CD4/CD8 ratios. The cumulative probability of achieving a CD4 count ≥ 800 cells/µl and/or CD4/CD8 ratio ≥ 1 over 10 years of ART were 54% for the CD4 threshold, 53% for the CD4/CD8 ratio threshold, and 32% for both thresholds. CD4 at baseline was identified as a predictor to achieve immune recovery in all models.

**Conclusions:**

Early diagnosis and treatment, as well as effective antiviral therapy without viral failure, should be considered to achieve long-term immune recovery among PWHIV.

**Supplementary Information:**

The online version contains supplementary material available at 10.1007/s15010-026-02740-y.

## Introduction

A hallmark of an untreated HIV infection is impaired immune function, immune stimulation, and an imbalance of immune cells. The number of CD4 cells, which coordinate adaptive immune responses, declines, while the number of CD8 cells, which support antiviral immune responses, increases [1]. Consequently, the CD4/CD8 ratio falls below 1 in most PWHIV [2].

Low CD4 cell counts are associated with an increased risk of AIDS-defining events among PWHIV [3]. Upon receiving antiretroviral therapy (ART), CD4 cell counts increase in most PWHIV and immune function can recover. PWHIV under ART can attain CD4 cell counts > 800 cells/µl, which approximated the median CD4 cell count in a sample of people without HIV [4–6]. Even among asymptomatic PWHIV with relatively well-preserved CD4 values, ART treatment and subsequent CD4 recovery can prevent long-term complications of HIV, such as the development of AIDS and non-AIDS related events [7, 8]. Earlier initiation of treatment increases the likelihood of CD4 cell recovery to higher levels while PWHIV with low CD4 counts were less likely to achieve this [9–11]. Consequently, current treatment guidelines in Germany recommend ART for all PWHIV, regardless of their immune status [12]. In addition, early diagnosis is also crucial to enable PWHIV to access effective treatment in time. Approximately one-third of new HIV diagnoses in 2024 are estimated to have occurred in PWHIV with severe immune defects or at the stage of AIDS [13]. There is a lack of long-term data on how many PWHIV in Germany manage to achieve good CD4 recovery while receiving long-term ART.

High CD8 cell counts and low CD4/CD8 ratios have been associated with reduced vaccine responsiveness, an increased risk of cancer, and higher mortality among PWHIV, even when virally suppressed and with CD4 values > 500 cells/µl [14–16]. Among PWHIV receiving ART, elevated CD8 cell counts can decrease slowly, and, together with increasing CD4 cells, the CD4/CD8 ratio can also increase [2]. Observational studies have shown that PWHIV with higher CD4 values and younger age at ART start were more likely to reach a normalised CD4/CD8 ratio of ≥ 1 [17, 18]. Moreover, women had a better recovery of CD4 cells and CD4/CD8 ratio compared to men, which might, however, not necessarily translate to similar improvements in mortality [19, 20]. Recent analyses have suggested that the combined use of CD4 values and the CD4/CD8 ratio might provide additional value in predicting long-term outcomes [14, 21]. PWHIV with CD4 values of at least 800 cells/µl, which approximates the median CD4 value among people without HIV, and a CD4/CD8 ratio of at least 1, which indicates a physiological proportion of immune cells, had a lower risk of developing AIDS during follow-up [4]. Moreover, a preserved immune system was suggested to contribute to favourable health outcomes also among people without HIV [4, 21]. There is a data gap concerning how many PWHIV under routine medical care and on permanent ART achieve immune recovery meeting these thresholds.

Long-term data on the development of CD4 cells in combination with CD4/CD8 ratios among PWHIV in routine medical care is scarce and studies are often difficult to conduct owing to limited observation time or PWHIV being lost to follow-up. In our analyses, we will investigate the long-term development of CD4 values and CD4/CD8 ratios over ten years. Furthermore, we will analyse the probability of PWHIV achieving the following immune thresholds: ≥ 800 CD4 cells/µl, a CD4/CD8 ratio of ≥ 1 and both thresholds together, adjusting for the probability of censoring. Lastly, we will identify predictors of achieving these thresholds.

## Methods

### Study design and setting

This study was conducted using data from two prospective German HIV cohorts. The HIV-1 Seroconverter study was initiated in 1997 and has been collecting data from HIV specialist practices and HIV outpatient clinics across Germany [22]. The study includes PWHIV aged ≥ 18 years, who have been diagnosed with HIV during acute seroconversion or within 3 years of a previous negative test result. The ClinSurv-HIV study was initiated in 1999 and has been collecting data from prevalent HIV PWHIV who are treated in clinical HIV centres across Germany [23]. In both cohort studies, baseline information on demographics, clinical status and ART is recorded upon recruitment. During follow-up, clinical and ART data are documented prospectively.

### Participant selection

In this analysis, we included ART-naive PWHIV aged 18 or older who initiated ART in 2003 or later. Follow-up started at the time of ART initiation and ended with the earliest occurrence of any of the following events: completion of 10 years of follow-up, treatment interruption for > 1 week, interruption of laboratory monitoring for > 6 months, loss to follow-up, death, or administrative censoring on 31 December 2018. Furthermore, PWHIV were censored if the ART they were receiving was not fully specified, or if they received monotherapy or treatment only with nucleoside reverse transcriptase inhibitors (NRTI). This occurred in 2% of PWHIV who were censored. In the time-to-event analyses, PWHIV were also censored upon experiencing the outcome. PWHIV with an undetectable viral load at baseline and without recorded ART were excluded from the analyses. In addition, PWHIV with less than two laboratory measurements or an observation period of less than 30 days were excluded. PWHIV were censored if their initial laboratory measurement occurred more than 100 days after ART start. PWHIV censored immediately at baseline (i.e., with no follow-up data) were excluded.

### Outcomes and variables

In our analyses, we investigated the absolute CD4 count in cells/µl and the probability of reaching CD4 thresholds of ≥ 800 cells/µl. Furthermore, we analysed the CD4/CD8 ratio and the probability of reaching a ratio of at least 1.

For our analyses, we selected one lab measurement containing absolute CD4 and CD8 values at baseline and every 6 month interval during the follow-up period, which extended up to 120 months. For each timepoint, all values within a 90 day period before or after that point were considered, and the laboratory measurement that fell closest to the timepoint was selected. Laboratory measurements missing information on either CD4 or CD8 were disregarded.

As covariables, we considered age at ART start in categories of 18–29, 30–39, 40–49, 50–59, and 60 + years, and sex as male or female. The transmission risk was categorised into the following groups: men who have sex with men (MSM); persons with heterosexual transmission (HET); people from high-prevalence countries (HPL); injecting drug users (IDU); other (e.g. mother-to-child transmission, haemophiliacs, transmission through blood products or occupational transmission); and unknown transmission route. The country or region of origin was coded as: Germany, Europe (excluding Germany), Africa / Middle East, North America / Australia / New Zealand, South America / Caribbean, Asia, and unknown. In the analyses of median CD4, the time-to-event analyses, and the predictor analyses, the country of origin was coded as a binary variable (Germany, other countries including unknown). The occurrence of death was recorded as yes/no. The year of ART start was analysed in categories of 2003–2007, 2008–2012, and 2013–2018. ART was analysed in categories of substance classes and the number of prescribed substances. Any combination of ART drugs was considered a regimen. If the class or the quantity of prescribed ART substances changed, the new combination was regarded as a new regimen. Conversely, a change of medication within the same substance class (e.g. an exchange of one protease-inhibitor against another) was not considered to be a regimen change. The occurrence of virological failure was defined as the presence of two consecutive viral load measurements > 200 copies/mL within a 100 day period among PWHIV who achieved viral suppression after starting ART.

### Statistical methods

A descriptive analysis was conducted on all variables, with medians and interquartile ranges utilised for continuous variables and absolute numbers and percentages for categorical variables. In instances where the transmission risk or country/region of origin was not known, this was categorised as “unknown” in a separate category.

In order to account for censoring over time, we calculated inverse probability censoring weights and used these in the time-to-event analyses [24]. In brief, we predicted the censoring probability of participants at each designated point in time on the basis of baseline variables, including transmission risk, age at ART start, CD4 count at ART start, and county/region of origin. Subsequently, we predicted the censoring probability using these baseline variables and the CD4 value from the previous time point. Next, we calculated stabilised weights for each timepoint by dividing the censoring probability from the model incorporating the CD4 value from the preceding timepoint by the censoring probability using only the baseline variables. We then multiplied all available probabilities for each person to derive one censoring weight per person. These weights were used in calculating Kaplan–Meier survival functions with the outcome of reaching the following immune thresholds: CD4 values ≥ 800 cells/µl, CD4/CD8 ratio ≥ 1 and the combination of both thresholds. Kaplan–Meier plots were calculated using locally weighted scatterplot smoothing (LOWESS).

In order to identify predictors for reaching the immune thresholds, a backward stepwise selection procedure with logistic regression was utilised. The potential predictors considered in this study included age at ART start, sex, transmission risk, country of origin, and CD4 count at ART start. Over 500 bootstrapping replications of the dataset, variables were retained in the model if their p-values was < 0.1. The number of models including the respective variables was then calculated. Variables being included in at least 80% of all bootstrapped models were considered as meaningful predictors.

We conducted a sensitivity analysis to assess the development of CD4 values among PWHIV who had completed all 10 years of follow-up (complete case analysis).

## Results

Of the 23,310 participants in both cohort studies, we included 8,927 ART-naive participants who met the following criteria: aged 18 years or over, initiated ART in 2003 or later, and had information on CD4 cell and CD4/CD8 measurements and ART at baseline (see Appendix 1 for details).

The median age at ART start was 39 years (interquartile range [IQR] 31–47) and most participants were male (Table 1). For the majority of participants, the estimated transmission risk was sex between men (53%). Other recorded risks included heterosexual transmission (17%), individuals from high-prevalence countries (12%), and intravenous drug use (4%). For 13% of the PWHIV, the probable route of HIV transmission was unknown.

**Table 1:** Baseline data

	PWHIV, n (%)
Total	8927
Observation time in years, median (IQR)	4.0 (1.7–7.5)
Age at ART start in years, median (IQR)	39 (31–47)
18–29, n (%)	1714 (19%)
30–39	2955 (33%)
40–49	2568 (29%)
50–59	1203 (13%)
60 +	487 (5%)
*Sex, n (%)*	
Male	7296 (82%)
Female	1631 (19%)
*HIV transmission risk, n (%)*	
MSM	4737 (53%)
HET	1523 (17%)
HPL	1112 (12%)
IDU	350 (4%)
Other	36 (0%)
Unknown	1169 (13%)
*Country/region of origin, n (%)*	
Germany	6218 (70%)
Europe	1022 (11%)
Africa / Middle East	1027 (12%)
North America / Australia / New Zealand	35 (0%)
South America / Caribbean	162 (2%)
Asia	292 (3%)
Unknown	171 (2%)
*Year of ART start, n (%)*	
2003 – 2007	2382 (27%)
2008 – 2012	3618 (41%)
2013 – 2018	2927 (33%)
CD4 at ART start, n (%)	
< 200	2607 (29%)
200–349	2667 (30%)
350–499	1864 (21%)
500–649	984 (11%)
650 +	805 (9%)
*CD8 at ART start, n(%)*	
> 1500	1723 (19%)
501 – 1500	5851 (66%)
≤ 500	1353 (15%)
*CD4/CD8 ratio at ART start, n (%)*	
≤ 1	8603 (96%)
> 1	324 (4%)
*Died, n (%)*	
No	8607 (96%)
Yes	320 (4%)
*Number of ART regimens during observation, n (%)*	
1	5770 (65%)
2	2110 (24%)
3	688 (8%)
4 +	359 (4%)
*Virological failure during observation, n (%)*	
None	8489 (95%)
1x	373 (4%)
≥ 2x	65 (1%)

*ART* antiretroviral therapy, *HET* heterosexual, *HPL* origin from high-prevalence country, *IDU* intravenous drug use, *IQR* interquartile range, *MSM* men, who have sex with men

More than two thirds of the participants were from Germany (Table 1). Other regions of origin included Africa/Middle East (12%) and Europe (11%). The majority of the PWHIV had baseline CD4 counts lower than 350 cells/µl and CD8 counts between 501 and 1500 cells/µl (Table 1). Only 4% of the PWHIV had a CD4/CD8 ratio ≥ 1 at ART start.

The median observation period was 4.0 years (IQR 1.7–7.5) with a minimum of 0.1 year and a maximum of 10.3 years. During the follow-up period, 4% of the PWHIV died. While the majority of PWHIV maintained the same ART regimen, 35% experienced at least one regimen change during follow-up (Table 1). Virological failure was observed at least once among 5% of the participants.

The median CD4 value at baseline was 257 cells/µl (Table 2). For PWHIV who remained under follow-up, this increased to 444 cells/µl after one year, 591 cells/µl after five years and 630 cells/µl after ten years of continuous ART. Median CD4 values were higher among younger PWHIV throughout follow-up (Table 2). Furthermore, male PWHIV had higher median CD4 values in comparison to female PWHIV at baseline and one year after ART initiation. However, after five years, the values became comparable and after ten years of follow-up, the median CD4 values were higher among women. With regard to transmission risk, PWHIV from high-prevalence countries had the lowest median CD4 values at baseline and throughout follow-up (Table 2). Conversely, MSM had the highest CD4 values at baseline and after one year of follow-up. After five years, the highest median CD4 values were observed among MSM and PWHIV with other transmission risks. After ten years, persons with injecting drug use and those with other transmission risks exhibited median CD4 cell counts above 700 cells/µl. CD4 counts were comparable regardless of whether PWHIV remained on their initial ART regimen or switched therapies. In contrast, CD4 values after 10 years were lower among PWHIV with 1 and 2 + more virological failures during follow up compared to PWHIV without virological failures.

**Table 2:** Median of CD4 cells among PWHIV under ART in Germany

	Median CD4 cells/µl (IQR)			
	At baseline	1 year after starting ART	5 years after starting ART	10 years after starting ART
Overall population in this observational period, n	8927	7461	3039	740
Median CD4 (IQR)	257 (124–393)	444 (290–622)	591 (430–760)	630 (474–811)
*Median CD4 by age at ART start (IQR)*				
18–29	305 (187–459)	519 (356–700)	672 (500–864)	696 (555–891)
30–39	259 (128–388)	449 (294–623)	600 (455–767)	659 (505–799)
40–49	243 (108–372)	426 (270–602)	598 (429–767)	635 (478–854)
50–59	215 (95–357)	400 (255–570)	521 (371–700)	569 (400–770)
60 +	230 (90–330)	379 (240–532)	478 (351–631)	505 (314–641)
*Median CD4 by sex (IQR)*				
Male	260 (127–398)	450 (298–629)	590 (428–759)	623 (470–797)
Female	237 (117–372)	403 (268–587)	592 (440–763)	675 (524–880)
*Median CD4 by HIV transmission risk (IQR)*				
MSM	284 (156–420)	480 (332–660)	619 (460–790)	654 (508–850)
HET	241 (111–381)	432 (278–607)	573 (420–730)	618 (462–784)
HPL	203 (89–323)	341 (220–496)	510 (361–701)	596 (445–751)
IDU	230 (115–351)	358 (231–545)	588 (416–741)	744 (621–937)
Other	251 (77–396)	412 (243–654)	656 (495–720)	749 (262–800)
Unknown	218 (84–365)	406 (257–600)	570 (399–740)	620 (459–796)
*Median CD4 by Country of origin (IQR)*				
Germany	266 (132–402)	460 (310–636)	600 (442–770)	630 (479–811)
Other / unknown countries	232 (110–370)	394 (253–585)	560 (399–732)	631 (458–811)
*Median CD4 by CD4 value at ART start (IQR)*				
< 200	73 (30–139)	239 (166–334)	425 (314–571)	530 (401–706)
200–349	250 (201–294)	410 (326–518)	588 (453–716)	658 (527–836)
350–499	377 (303–429)	546 (450–660)	700 (570–862)	743 (578–889)
500–649	515 (381–578)	685 (570–824)	827 (665–967)	790 (644–1008)
650 +	702 (543–814)	892 (718–1062)	1003 (753–1235)	930 (775–1300)
*Median CD4 by number of ART regimens during observation (IQR)*				
1	270 (143–409)	456 (306–633)	596 (437–780)	632 (502–814)
2	234 (102–376)	422 (270–605)	576 (407–743)	616 (442–791)
3	228 (82–352)	440 (270–602)	597 (430–730)	656 (468–848)
4 +	211 (95–342)	403 (287–573)	616 (485–791)	684 (504–875)
*Median CD4 by virological failure during observation (IQR)*				
None	260 (130–398)	450 (298–630)	596 (438–766)	641 (497–817)
1x	167 (60–281)	341 (215–489)	497 (359–691)	483 (360–725)
≥ 2x	167 (89–251)	279 (180–410)	500 (275–626)	525 (490–679)

*ART* antiretroviral therapy, *HET* heterosexual, *HPL* origin from high-prevalence country, *IDU* intravenous drug use, *IQR* interquartile range, *MSM* men, who have sex with men

The median CD4/CD8 ratio at baseline was 0.26 and increased to 0.84 after 10 years of follow-up. CD4/CD8 ratios were found to be consistently higher among the youngest age group (Table 3). While the CD4/CD8 ratio was comparable among men and women at baseline and after one year, women exhibited a higher ratio after years 5 and 10 (Table 3). PWHIV with higher CD4 values at baseline also had consequently higher ratios throughout follow-up with median CD4/CD8 ratios exceeding 1 after five and ten years among PWHIV with ≥ 650 CD4 cells/µl at baseline. Similarly, PWHIV with lower CD8 values at baseline also had higher CD4/CD8 ratios at both baseline and during follow-up. While the distribution of the CD4/CD8 ratio appeared comparable among PWHIV with and without ART changes during follow-up, it was lower among PWHIV who had one or more instances of virological failure.

**Table 3:** Median of CD4/CD8 ratio among PWHIV under ART in Germany

	Median CD4/CD8 ratio (IQR)			
	At baseline	1 year after starting ART	5 years after starting ART	10 years after starting ART
Overall population in this observational period, n	8927	7461	3039	740
Median CD4/CD8 ratio (IQR)	0.26 (0.14–0.43)	0.52 (0.32–0.78)	0.75 (0.52–1.05)	0.84 (0.61–1.11)
*Median CD4/CD8 ratio by age at ART start (IQR)*				
18–29	0.33 (0.20–0.51)	0.64 (0.43–0.89)	0.85 (0.65–1.10)	0.95 (0.72–1.29)
30–39	0.26 (0.15–0.42)	0.50 (0.33–0.76)	0.74 (0.55–1.03)	0.86 (0.63–1.09)
40–49	0.24 (0.13–0.39)	0.49 (0.29–0.74)	0.75 (0.50–1.06)	0.80 (0.59–1.03)
50–59	0.23 (0.12–0.39)	0.47 (0.26–0.74)	0.70 (0.44–1.05)	0.83 (0.57–1.29)
60 +	0.22 (0.12–0.37)	0.45 (0.27–0.73)	0.66 (0.44–0.97)	0.69 (0.46–1.02)
*Median CD4/CD8 ratio by sex (IQR)*				
Male	0.25 (0.14–0.42)	0.51 (0.31–0.77)	0.73 (0.51–1.03)	0.82 (0.59–1.07)
Female	0.28 (0.14–0.46)	0.54 (0.33–0.84)	0.84 (0.59–1.15)	0.97 (0.76–1.31)
*Median CD4/CD8 ratio by HIV transmission risk (IQR)*				
MSM	0.27 (0.16–0.43)	0.54 (0.35–0.79)	0.75 (0.54–1.04)	0.82 (0.60–1.06)
HET	0.26 (0.14–0.44)	0.56 (0.33–0.86)	0.81 (0.55–1.18)	0.88 (0.63–1.31)
HPL	0.23 (0.11–0.39)	0.41 (0.24–0.67)	0.71 (0.48–0.96)	0.88 (0.63–1.07)
IDU	0.27 (0.15–0.42)	0.47 (0.26–0.71)	0.72 (0.49–1.04)	0.87 (0.76–1.07)
Other	0.31 (0.14–0.51)	0.50 (0.27–0.92)	0.90 (0.70–1.33)	0.82 (0.53–1.67)
Unknown	0.23 (0.12–0.39)	0.48 (0.27–0.76)	0.74 (0.48–1.01)	0.79 (0.60–1.13)
*Median CD4/CD8 ratio by Country of origin (IQR)*				
Germany	0.26 (0.15–0.42)	0.53 (0.33–0.79)	0.76 (0.53–1.06)	0.82 (0.60–1.12)
Other / unknown countries	0.26 (0.13–0.44)	0.48 (0.29–0.76)	0.74 (0.51–1.03)	0.88 (0.61–1.06)
*Median CD4/CD8 ratio by CD4 value at ART start (IQR)*				
< 200	0.11 (0.05–0.20)	0.28 (0.18–0.42)	0.53 (0.37–0.76)	0.71 (0.49–0.98)
200–349	0.25 (0.17–0.36)	0.51 (0.35–0.72)	0.78 (0.58–1.03)	0.90 (0.69–1.13)
350–499	0.34 (0.23–0.48)	0.65 (0.47–0.88)	0.88 (0.68–1.19)	0.96 (0.72–1.24)
500–649	0.42 (0.29–0.58)	0.74 (0.56–1.01)	1.00 (0.72–1.31)	0.83 (0.67–1.27)
650 +	0.55 (0.36–0.79)	0.93 (0.68–1.31)	1.20 (0.87–1.56)	1.32 (0.85–1.65)
*Median CD4/CD8 ratio by CD8 value at ART start (IQR)*				
> 1500	0.17 (0.11–0.26)	0.40 (0.25–0.58)	0.64 (0.45–0.83)	0.72 (0.51–0.94)
501–1500	0.29 (0.17–0.45)	0.56 (0.35–0.82)	0.78 (0.55–1.09)	0.86 (0.64–1.12)
≤ 500	0.29 (0.10–0.58)	0.51 (0.28–0.91)	0.80 (0.54–1.17)	0.92 (0.67–1.30)
*Median CD4/CD8 ratio by number of ART regimens during observation (IQR)*				
1	0.27 (0.15–0.45)	0.54 (0.33–0.80)	0.77 (0.54–1.08)	0.88 (0.64–1.14)
2	0.24 (0.13–0.39)	0.48 (0.28–0.73)	0.72 (0.51–1.00)	0.82 (0.58–1.07)
3	0.23 (0.12–0.39)	0.50 (0.29–0.75)	0.75 (0.50–1.04)	0.82 (0.61–1.09)
4 +	0.22 (0.12–0.35)	0.45 (0.28–0.65)	0.76 (0.51–1.07)	0.78 (0.61–1.13)
*Median CD4/CD8 ratio by virological failure during observation (IQR)*				
None	0.26 (0.15–0.43)	0.53 (0.33–0.79)	0.76 (0.53–1.06)	0.86 (0.63–1.12)
1x	0.19 (0.09–0.32)	0.35 (0.21–0.58)	0.61 (0.40–0.87)	0.66 (0.49–0.95)
≥ 2x	0.20 (0.12–0.27)	0.33 (0.21–0.47)	0.54 (0.35–0.89)	0.50 (0.34–0.61)

*ART* antiretroviral therapy, *HET* heterosexual, *HPL* origin from high-prevalence country, *IDU* intravenous drug use, *IQR* interquartile range, *MSM* men, who have sex with men

Throughout the observation period, the majority of PWHIV (8,187/8,927) were censored at some point (Tables 2 and 3). Almost all of these PWHIV (98%) were censored due to missing longitudinal data, while 2% were censored due to the prescribed ART regimens (see methods section). When the sample was restricted to the 740 PWHIV with complete 10 year follow-up data, similar trends in CD4 and CD4/CD8 ratio development were observed (Appendix 2 and 3). Furthermore, an analysis was conducted to ascertain whether the distribution of the baseline variables changed among PWHIV included during different timepoints due to selective drop-out. The characteristics by sex, transmission risk and country/region of origin appeared stable over time (Appendix 4). However, the proportion of PWHIV with low CD4 values at baseline increased while the proportion of PWHIV with younger age at ART start decreased. In order to adjust for the potential effects of censoring, inverse probability censoring weights were calculated for all PWHIV in the dataset as described. The minimum weight was at 0.504, while the maximum was 1.254, with a mean of 1.001 (Appendix 5). These weights were utilised in the following analyses as probability weights.

**Fig. 1:**
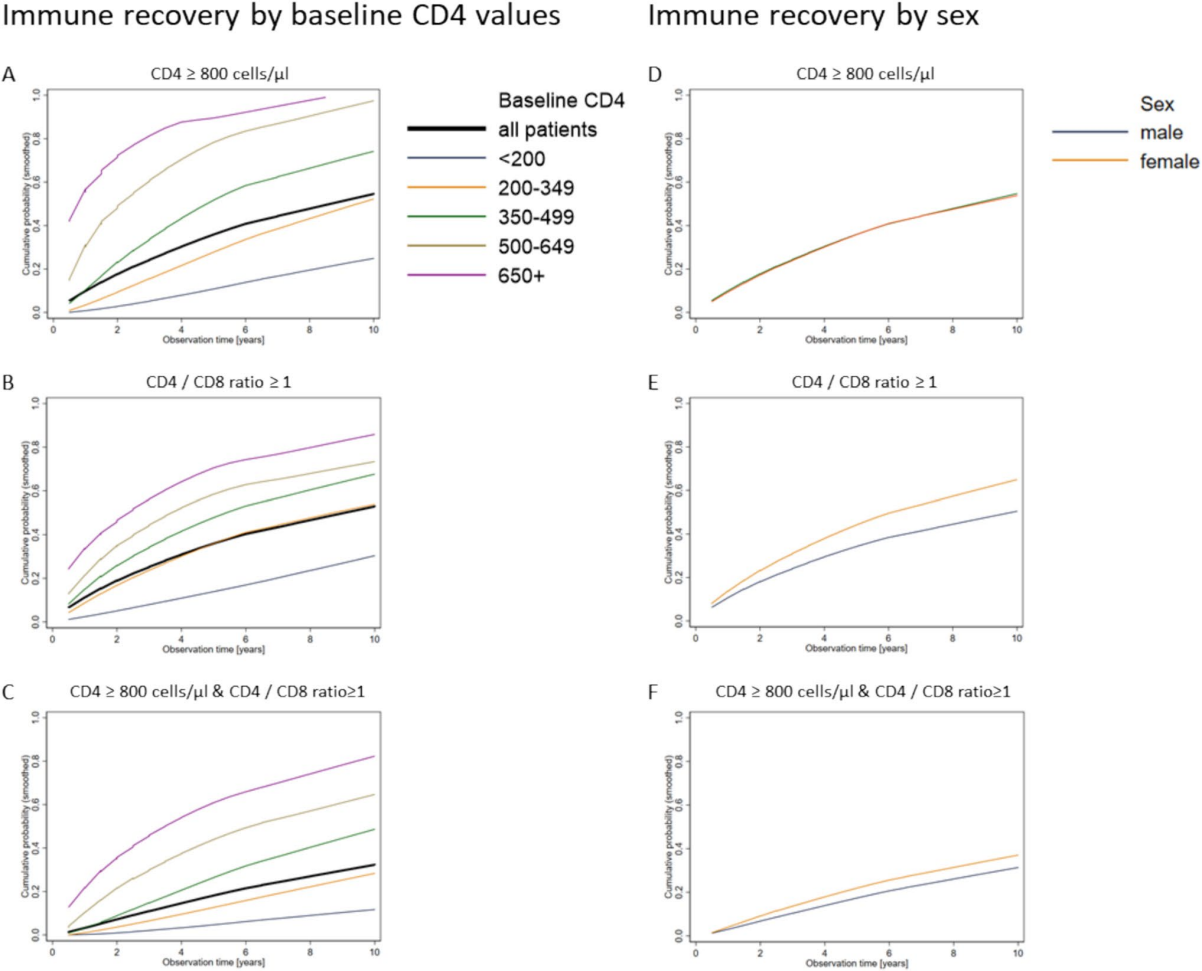
Cumulative probability (smoothed) of reaching CD4 values of ≥ 800 cells/ (n = 8031) (**A**, **D**), a CD4/CD8 ratio ≥ 1 (n = 8054 (**B**, **E**)), and both CD4 ≥ 800 cells/µl and CD4/CD8 ratio ≥ 1 (n = 7848) (**C**, **F**), stratified by CD4 at baseline (**A**–**C**) and sex (**D**–**F**)

Three Kaplan–Meier analyses were performed to assess the time to reach CD4 counts of ≥ 800 cells/µl, a CD4/CD8 ratio of ≥ 1, and the combination of these two thresholds. In the first analysis, the study population included 8031 PWHIV with CD4 values below 800 cells/µl at baseline, who contributed person-time. Overall, 54% of the PWHIV managed to reach this threshold at some point over the 10 years of follow-up. While almost all PWHIV with baseline CD4 values ≥ 500 were able to attain this threshold, this was the case for only approximately half of the PWHIV with baseline CD4 values of 200–349 cells/µl (Fig. 1A). In PWHIV with a CD4 cell count of less than 200 cells/µl, only about 1 in 4 demonstrated an increase to at least 800 CD4 cells/µl over ten years of ART.

For the analyses of reaching a CD4/CD8 ratio ≥ 1, we included 8054 PWHIV who started observation with a ratio of less than 1 and contributed person-time to the analysis. The probability of achieving immune recovery as indicated by a CD4/CD8 ratio ≥ 1 after 10 years was 53% and in a similar range as the CD4 recovery alone. Moreover, it was also contingent on baseline CD4 values (Fig. 1B). However, the proportion of PWHIV with high baseline CD4 values reaching the ratio threshold was lower compared to PWHIV reaching the CD4 threshold. Consequently, about 1 in 5 PWHIV with baseline CD4 counts of ≥ 650 cells/µl did not attain a CD4/CD8 ratio ≥ 1.

Regarding the probability of reaching the combined thresholds CD4 ≥ 800 and CD4/CD8 ratio ≥ 1, 32% reached this threshold over 10 years (Fig. 1C). While more than half of the PWHIV with a CD4 baseline value of at least 500 cells/µl managed to achieve this level of immune recovery, this was observed for only about a quarter of PWHIV with baseline CD4 values of 200–349 cells/µl. A similar pattern was observed among PWHIV with even lower baseline CD4 values. Moreover, women were more likely to achieve the CD4/CD8 ratio threshold and combined threshold (CD4 ≥ 800 and CD4/CD8 ratio ≥ 1) compared with men (Fig. 1E and F). The proportion of PWHIV attaining the CD4 and CD4/CD8 ratio thresholds continued to increase throughout the observational period of 10 years.

We then identified predictors for attaining the threshold of ≥ 800 CD4 cells/µl, a CD4/CD8 ratio ≥ 1, or both thresholds together. Over 500 bootstrapping replications for each outcome, logistic regression models were constructed using a backward modelling approach, retaining variables with p < 0.1. We then counted, how often variables were included in the models and considered variables included in ≥ 400 (≥ 80%) of all models as meaningful predictors. The inclusion frequencies are displayed in Table 4. CD4 count at baseline was included in all models, indicating that higher CD4 count at baseline was of high predictive importance for all three outcome variables. Age met the threshold as a meaningful predictor for the outcome of reaching of ≥ 800 CD4 cells/µl (included in 400/500 models), but not for the outcomes of the CD4/CD8 ratio ≥ 1 (included in 48/500 models) and both thresholds (included in 316/500 models). Conversely, sex was included in the model in all bootstrapped samples in the analyses of the outcome CD4/CD8 ratio ≥ 1 as well as both thresholds simultaneously replicating the findings of Kaplan–Meier analyses (Fig. 1) and suggesting strong predictive utility for these outcomes. Country of origin was identified as a predictor for the outcomes of ≥ 800 CD4 cells/µl or a CD4/CD8 ratio ≥ 1, but not for reaching both thresholds simultaneously. HIV transmission risk was not identified as a meaningful predictor for any of the three outcomes.

**Table 4:** Inclusion frequencies of variables in predictive models for reaching immunological thresholds after 500 bootstrap replications

	Reaching CD4 ≥ 800 cells/µl	Reaching CD4/CD8 ratio ≥ 1	Reaching CD4 ≥ 800 cells/µl & CD4/CD8 ratio ≥ 1)
Number of included PWHIV	8031	8054	7848
Age at ART start (continuous)	400	48	316
Sex	290	500	500
HIV transmission risk	78	97	60
Country of origin (binary)	476	496	340
CD4 at ART start	500	500	500

Results of 500 bootstrapping replications per investigated outcomes. For each replication, variables were retained in the model if their p-value was < 0.1. The number of models in which the variables were included is displayed in the table. Variables included in ≥ 80% of the models were considered as predictors

## Discussion

Over ten years of follow-up, we observed strong improvements in median CD4 cell counts (from 257 to 630 cells/µl) and in the CD4/CD8 ratio (from 0.26 to 0.84) among people receiving continuous ART. Recovery of both parameters was higher in younger PWHIV, female PWHIV, PWHIV with higher baseline CD4 values, and PWHIV without viral failure under ART. Over ten years, 54% attained a CD4 count ≥ 800 cells/µl, 53% a CD4/CD8 ratio ≥ 1, and 32% attained both thresholds. CD4 at baseline was a strong predictor of achieving immune recovery for all three outcomes.

In our analysis, the median CD4 value after 10 years of ART was 630 cells/µl (IQR 474–811). This is comparable to an analysis from a London cohort, where the median CD4 cell count was 663 cells/µl after 10 years [25]. Furthermore, a model based on data from the Swiss Cohort Study suggested that the CD4 plateau under long-term ART would be around 718 cells/µl [26]. While this is higher than what we observed in our study, it is plausible that CD4 recovery continues beyond ten years of ART. We also observed that 54% of the PWHIV achieved a CD4 cell count of at least 800 cells/µl at some point during follow-up. This is comparable to a ten-year observation from a Polish cohort, which found that 48.1% of their PWHIV reached this immune threshold after ten years of ART [5]. Other studies looked at reaching even higher CD4 thresholds and found that recovery to at least 900 CD4 cells occurred among only in 18.7% of the PWHIV [17]. However, the median follow-up duration was shorter than in our study, which may have impacted the results.

Furthermore, an analysis of PWHIV who were treated within four months of their estimated date of infection revealed that 64% of these PWHIV achieved a recovery to at least 900 CD4 cells within 48 months [10]. This suggests that early treatment provides a better chance of achieving higher CD4 recovery levels, which is consistent with our finding that PWHIV with higher baseline CD4 values – often indicating a shorter period of untreated HIV infection – were also more likely to achieve CD4 recovery to 800 cells/µl. Similarly, an analysis from the ART-CC cohort found that median CD4 values after eight years of ART were also highly dependent on the baseline CD4 cell count [18]. Taken together, these results strongly suggest that early diagnosis and treatment of HIV are important components for immune recovery.

We also observed that the median CD4 count was lower by more than 100 cells/µl among PWHIV who experienced viral failure during ART. This aligns with data from Asian and Australian HIV cohorts, in which PWHIV who experienced viral failure within the first 10 years of ART also had an average lower CD4 count of 79 cells/µl [27]. Thus, effective ART also appears to be an important contributor to CD4 cell recovery.

In addition to observing CD4 recovery, we also analysed how the CD4/CD8 ratio developed over time. The median ratio after 10 years was 0.84 (IQR 0.61–1.11) in our cohort, similar to the median the CD4/CD8 ratio after 10 years of ART reported in a London cohort, which was 0.88 (IQR 0.64–1.17) [25]. Analysis of data from the ART-CC cohort revealed a median CD4/CD8 ratio of 0.81 (IQR 0.57–1.11) after eight years of ART [18]. In our Kaplan–Meier analyses using inverse probability censoring weights, 53% of PWHIV attained a normalised ratio over the observational period. This is similar to the results from a Polish cohort, in which 48.3% of the PWHIV reached a CD4/CD8 ratio ≥ 1 over 10 years of follow-up [5]. Modelling of CD4/CD8 ratio development over 10 years of follow-up suggested a median plateau of 1.01, predicting that approximately half of the PWHIV eventually achieve a normalised CD4/CD4 ratio [26]. In contrast, another study found that only 31.3% of PWHIV achieved a normalised ratio; however, the shorter observation period may also have contributed to this lower proportion [17].

Starting ART early supports the normalisation of the CD4/CD8 ratio. A Brazilian study showed that over half of the PWHIV treated during acute or recent HIV infection had a normalised CD4/CD8 ratio after one year of treatment [28]. In contrast, only 8% of the PWHIV with a chronic HIV infection and CD4 counts < 350 cells/µl achieved a normalised CD4/CD8 ratio after one year of ART, rising to 27% after four years. Earlier treatment was also linked to better CD4/CD8 ratio improvement in the model for long-term immune recovery [26]. While our dataset does not include information on time of infection, baseline CD4 levels can approximate the duration of an untreated HIV infection. Our findings show that PWHIV with higher baseline CD4 values, indicating a shorter duration of infection, were more likely to achieve normalised CD4/CD8 ratios. This association between higher CD4 cell counts at baseline and a higher likelihood of achieving normalised CD4/CD8 ratios has also been observed in other studies [17, 18]. Furthermore, PWHIV with lower CD8 cell counts at baseline, and thus a higher CD4/CD8 ratio, were also more likely to achieve normalised ratios [18].

PWHIV who experienced one or more viral failures during ART treatment had lower CD4/CD8 ratios during follow-up than those who achieved complete viral suppression. This was also observed in a Mexican cohort, where PWHIV with better ART adherence had higher CD4/CD8 ratios [29]. Therefore, effective treatment also plays an important role in the recovery of CD4/CD8 ratios. The discrepancy between observing impaired CD4/CD8 ratios among people with virological failure but not among those with ART switches could likely be the result of switching in the absence of virological failure. As shown in Table 1, only 5% of PWHIV experienced at least one event of virological failure whereas 35% underwent one or more switches in therapy regimens. Thus, the majority of PWHIV will have experienced switches because of other reasons, e.g. treatment simplification or intolerance/toxicity.

Previous studies have shown that combining the two thresholds of a CD4 cell count of at least 800 cells/µl and CD4/CD8 ratio of at least 1.0 can be a meaningful indicator of immune recovery and immunological resilience [4, 14, 21]. Maintenance of immune function as defined by both thresholds was associated with a lower risk of progression to AIDS among PWHIV [4]. Moreover, it was linked to better survival among people without HIV with other diseases, such as COVID-19. In our study, 32% had achieved immune recovery to both thresholds over ten years of ART. This is consistent with data from a Polish cohort, in which 34.7% achieved this outcome after ten years [5]. Among PWHIV with a primary HIV infection, 46% achieved this outcome after just four years [4]. Early treatment after primary HIV infection appears to drive the increased levels of immune recovery in this cohort.

The results of our study and the published evidence showed that earlier treatment of HIV infection can lead to better long-term immune recovery regarding both CD4 values and CD4/CD8 ratios. Therefore, early HIV diagnosis must remain a key objective of public health strategies. A systematic review estimated the mean time between infection and diagnosis at three years, which was shorter among MSM than people with other transmission risks [30]. Moreover, a substantial number of people receive their diagnosis at the stage of AIDS or with advanced immune suppression [13]. Another study showed that people with heterosexual HIV transmission were more likely to be diagnosed with a chronic HIV infection or at the stage of AIDS than MSM [31]. Therefore, efforts to ensure earlier diagnosis of PWHIV in Germany across all people at risk of HIV transmission are needed. We also demonstrated that virological failure during ART treatment impaired immune recovery over time. Therefore, the risk of virological failure should be minimised by selecting effective ART regimens that facilitate adherence [32].

Although not all PWHIV will achieve immune recovery during continuous ART treatment, monitoring of both surrogate markers—the CD4 cell count and CD4/CD8 ratio—over time may still be useful. PWHIV with low immune recovery might benefit from more intensive screening for cancers such as e.g. anal cancer, non-Hodgkin lymphoma and lung cancer [15, 33, 34]. Another study showed that low CD4 values before viral suppression were associated with increased rates of hospitalisations for up to 11 years of follow-up [35]. Therefore, PWHIV with impaired immune systems before and during ART should undergo closer monitoring for potential long-term complications.

The major strength of our study is the long observation period together with the accounting for censoring, which overcomes some limitations of previous analyses. Furthermore, the large study population enabled meaningful analyses and the characterisation of subgroups. However, some limitations also need to be considered. Although we accounted for the probability of censoring over time based on the specified variables, it is possible that differential censoring based on an unmeasured factor might have affected the results. However, the results of our sensitivity analyses and of other published studies are comparable to our main results, and we currently have no evidence on an unmeasured influencing factor. Another limitation is that we could not stratify the results by duration of infection before starting ART, since this information was not available in the dataset. Therefore, we had to rely on baseline CD4 values to approximate the duration of HIV infection. However, some PWHIV who were diagnosed early could also have presented with low CD4 values, meaning that the baseline values can only serve as a rough approximate [36].

## Conclusion

The long-term immune recovery of PWHIV over more than ten years differed according to their characteristics. During the first ten years of ART, around half of the PWHIV achieved a CD4 count of at least 800 cells/µl or a CD4/CD8 ratio of at least 1, but only around a third attained both thresholds. Early treatment with ART and high adherence to avoid virological failure appear to be important contributors to achieve immune recovery. PWHIV with impaired immune recovery should receive enhanced clinical monitoring for possible complications, such as cancer.

## Supplementary Information

Below is the link to the electronic supplementary material.

Supplementary file1 (DOCX 184 KB)

## Data Availability

The dataset from this study is not publicly available due to data protection and confidentiality agreements.
