# Supplementary material for: Long-term immune recovery under continuous antiretroviral therapy (ART) among ART-naive people living with HIV in two cohorts in Germany
Source: Infection. 2026 Feb 26;54(2):959–70. doi: 10.1007/s15010-026-02740-y (PMC13021689; doi:10.1007/s15010-026-02740-y)

**Long-term immune recovery under continuous ART among therapy-naive people living with HIV in two HIV cohorts in Germany 2003 – 2018 – Appendix**

Appendix 1: Participant selection


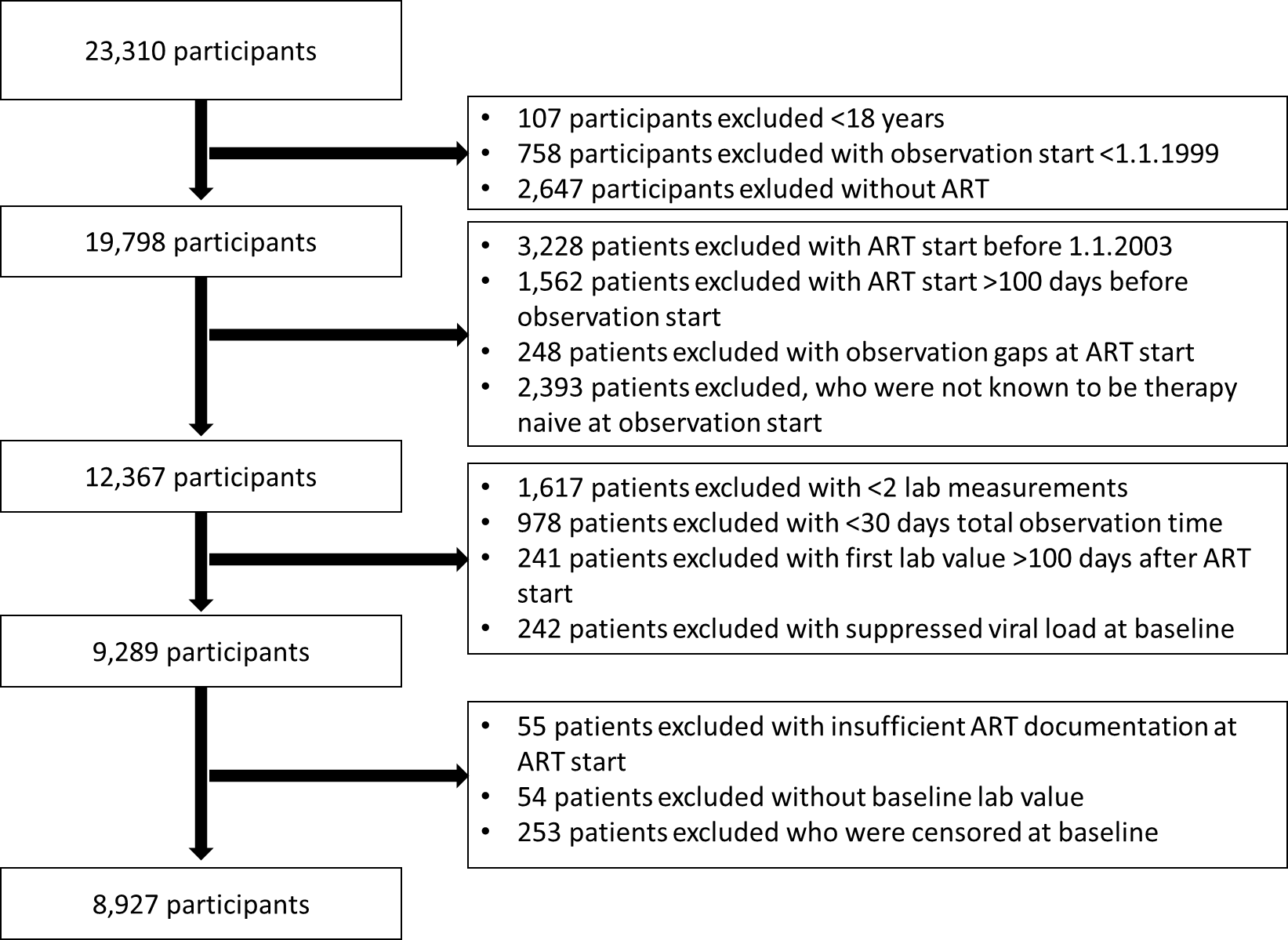


Appendix 2: Median of CD4 among PWHIV under ART in Germany restricted to patients with complete observation

|  | Median CD4 cells/µl (IQR) | | | |
| --- | --- | --- | --- | --- |
|  | **At baseline** | **1 year after starting ART** | **5 years after starting ART** | **10 years after starting ART** |
| Overall population in this observational period, n | 740 | 740 | 740 | 740 |
| Median CD4 (IQR) | 195 (70 - 290) | 376 (250 - 515) | 546 (404 - 715) | 630 (474 - 811) |
| Median CD4 by age at ART start, median (IQR) |  |  |  |  |
| 18-29 | 205 (132 - 301) | 432 (298 - 629) | 590 (472 - 797) | 696 (555 - 891) |
| 30-39 | 189 (74 - 280) | 374 (262 - 513) | 559 (426 - 718) | 659 (505 - 799) |
| 40-49 | 205 (65 - 301) | 379 (245 - 515) | 555 (410 - 720) | 635 (478 - 854) |
| 50-59 | 158 (62 - 286) | 320 (229 - 473) | 462 (328 - 666) | 569 (400 - 770) |
| 60+ | 178 (58 - 285) | 341 (224 - 486) | 456 (313 - 619) | 505 (314 - 641) |
| Median CD4 by sex, median (IQR) |  |  |  |  |
| Male | 199 (70 - 302) | 378 (250 - 516) | 547 (401 - 710) | 623 (470 - 797) |
| Female | 182 (74 - 242) | 360 (255 - 514) | 541 (426 - 742) | 675 (524 - 880) |
| Median CD4 by HIV transmission risk, median (IQR) |  |  |  |  |
| MSM | 210 (87 - 320) | 398 (286 - 532) | 575 (433 - 740) | 654 (508 - 850) |
| HET | 196 (66 - 297) | 387 (254 - 530) | 514 (372 - 674) | 618 (462 - 784) |
| HPL | 160 (63 - 239) | 300 (187 - 429) | 511 (366 - 677) | 596 (445 - 751) |
| IDU | 180 (128 - 253) | 382 (300 - 445) | 565 (422 - 648) | 744 (621 - 937) |
| Other | 69 (8 - 286) | 438 (81 - 449) | 452 (261 - 606) | 749 (262 - 800) |
| Median CD4 by CD4 value at ART start, median (IQR) |  |  |  |  |
| <200 | 66 (28 - 137) | 244 (161 - 336) | 437 (294 - 570) | 530 (401 - 706) |
| 200-349 | 255 (206 - 290) | 408 (328 - 517) | 589 (455 - 715) | 658 (527 - 836) |
| 350-499 | 357 (222 - 394) | 514 (421 - 634) | 641 (509 - 800) | 743 (578 - 889) |
| 500-649 | 444 (336 - 524) | 655 (513 - 760) | 819 (624 - 919) | 790 (644 - 1008) |
| 650+ | 655 (463 - 811) | 957 (708 - 1159) | 850 (688 - 1146) | 930 (775 - 1300) |
| Median CD4 by number of ART regimens during observation, median (IQR) |  |  |  |  |
| 1 | 203 (97 - 299) | 381 (263 - 528) | 541 (410 - 720) | 632 (502 - 814) |
| 2 | 193 (57 - 290) | 361 (240 - 494) | 536 (393 - 710) | 616 (442 - 791) |
| 3 | 174 (55 - 276) | 366 (217 - 527) | 550 (397 - 675) | 656 (468 - 848) |
| 4+ | 182 (73 - 284) | 385 (265 - 527) | 607 (491 - 760) | 684 (504 - 875) |
| Median CD4 by virological failure during observation, median (IQR) |  |  |  |  |
| None | 200 (74 - 294) | 381 (262 - 524) | 553 (417 - 720) | 641 (497 - 817) |
| 1x | 131 (46 - 245) | 303 (190 - 428) | 466 (314 - 657) | 483 (360 - 725) |
| ≥2x | 156 (89 - 208) | 226 (134 - 360) | 514 (266 - 603) | 525 (490 - 679) |

Appendix 3: Median of CD4/CD8 ratio among PWHIV under ART in Germany restricted to patients with complete observation

|  | Median CD4/CD8 ratio (IQR) | | | |
| --- | --- | --- | --- | --- |
|  | **At baseline** | **1 year after starting ART** | **5 years after starting ART** | **10 years after starting ART** |
| Overall population in this observational period, n | 740 | 740 | 740 | 740 |
| Median CD4/CD8 ratio (IQR) | 0.19 (0.11 - 0.30) | 0.42 (0.26 - 0.63) | 0.69 (0.48 - 0.95) | 0.84 (0.61 - 1.11) |
| Median CD4/CD8 ratio by age at ART start, median (IQR) |  |  |  |  |
| 18-29 | 0.24 (0.16 - 0.35) | 0.52 (0.38 - 0.77) | 0.82 (0.63 - 1.04) | 0.95 (0.72 - 1.29) |
| 30-39 | 0.17 (0.09 - 0.29) | 0.42 (0.25 - 0.61) | 0.69 (0.48 - 0.93) | 0.86 (0.63 - 1.09) |
| 40-49 | 0.20 (0.10 - 0.31) | 0.39 (0.25 - 0.62) | 0.69 (0.45 - 0.95) | 0.80 (0.59 - 1.03) |
| 50-59 | 0.22 (0.11 - 0.31) | 0.40 (0.24 - 0.69) | 0.65 (0.43 - 1.10) | 0.83 (0.57 - 1.29) |
| 60+ | 0.17 (0.09 - 0.30) | 0.41 (0.25 - 0.57) | 0.58 (0.39 - 0.87) | 0.69 (0.46 - 1.02) |
| Median CD4/CD8 ratio by sex, median (IQR) |  |  |  |  |
| Male | 0.19 (0.10 - 0.31) | 0.42 (0.26 - 0.63) | 0.67 (0.47 - 0.94) | 0.82 (0.59 - 1.07) |
| Female | 0.19 (0.11 - 0.30) | 0.44 (0.29 - 0.64) | 0.78 (0.55 - 1.09) | 0.97 (0.76 - 1.31) |
| Median CD4/CD8 ratio by HIV transmission risk, median (IQR) |  |  |  |  |
| MSM | 0.20 (0.12 - 0.31) | 0.44 (0.28 - 0.63) | 0.69 (0.50 - 0.94) | 0.82 (0.60 - 1.06) |
| HET | 0.21 (0.10 - 0.31) | 0.48 (0.25 - 0.74) | 0.71 (0.51 - 1.14) | 0.88 (0.63 - 1.31) |
| HPL | 0.16 (0.07 - 0.27) | 0.35 (0.22 - 0.48) | 0.64 (0.47 - 0.88) | 0.88 (0.63 - 1.07) |
| IDU | 0.31 (0.19 - 0.48) | 0.53 (0.33 - 0.74) | 0.72 (0.52 - 1.04) | 0.87 (0.76 - 1.07) |
| Other | 0.12 (0.02 - 0.31) | 0.41 (0.10 - 0.67) | 0.58 (0.47 - 1.40) | 0.82 (0.53 - 1.67) |
| Median CD4/CD8 ratio by CD4 value at ART start, median (IQR) |  |  |  |  |
| <200 | 0.11 (0.05 - 0.20) | 0.26 (0.18 - 0.42) | 0.53 (0.37 - 0.77) | 0.71 (0.49 - 0.98) |
| 200-349 | 0.23 (0.16 - 0.31) | 0.49 (0.35 - 0.67) | 0.74 (0.58 - 1.03) | 0.90 (0.69 - 1.13) |
| 350-499 | 0.25 (0.18 - 0.39) | 0.52 (0.41 - 0.78) | 0.82 (0.64 - 1.06) | 0.96 (0.72 - 1.24) |
| 500-649 | 0.30 (0.19 - 0.44) | 0.60 (0.45 - 0.74) | 0.81 (0.67 - 1.01) | 0.83 (0.67 - 1.27) |
| 650+ | 0.55 (0.23 - 0.83) | 0.77 (0.58 - 1.48) | 1.26 (0.79 - 1.81) | 1.32 (0.85 - 1.65) |
| Median CD4/CD8 ratio by CD8 value at ART start (IQR) |  |  |  |  |
| > 1500 | 0.15 (0.08 - 0.19) | 0.35 (0.21 - 0.48) | 0.58 (0.39 - 0.80) | 0.72 (0.51 - 0.94) |
| 501 – 1500 | 0.22 (0.13 - 0.31) | 0.45 (0.29 - 0.67) | 0.71 (0.51 - 0.98) | 0.86 (0.64 - 1.12) |
| ≤ 500 | 0.21 (0.07 - 0.41) | 0.44 (0.26 - 0.72) | 0.75 (0.49 - 1.05) | 0.92 (0.67 - 1.30) |
| Median CD4/CD8 ratio by number of ART regimens during observation, median (IQR) |  |  |  |  |
| 1 | 0.20 (0.12 - 0.31) | 0.44 (0.29 - 0.67) | 0.71 (0.50 - 0.98) | 0.88 (0.64 - 1.14) |
| 2 | 0.19 (0.08 - 0.28) | 0.39 (0.24 - 0.62) | 0.67 (0.47 - 0.92) | 0.82 (0.58 - 1.07) |
| 3 | 0.16 (0.08 - 0.28) | 0.40 (0.23 - 0.55) | 0.68 (0.48 - 0.96) | 0.82 (0.61 - 1.09) |
| 4+ | 0.20 (0.12 - 0.35) | 0.47 (0.29 - 0.67) | 0.70 (0.45 - 1.03) | 0.78 (0.61 - 1.13) |
| Median CD4/CD8 ratio by virological failure during observation, median (IQR) |  |  |  |  |
| None | 0.20 (0.11 - 0.31) | 0.44 (0.27 - 0.64) | 0.70 (0.49 - 0.97) | 0.86 (0.63 - 1.12) |
| 1x | 0.16 (0.06 - 0.24) | 0.32 (0.22 - 0.58) | 0.62 (0.38 - 0.83) | 0.66 (0.49 - 0.95) |
| ≥2x | 0.19 (0.06 - 0.24) | 0.26 (0.20 - 0.33) | 0.50 (0.35 - 0.70) | 0.50 (0.34 - 0.61) |

Appendix 4: Distribution of baseline characteristics among patients still under follow up over the observation period

Appendix 5: Distribution of inverse probability censoring weights for all patients in the dataset

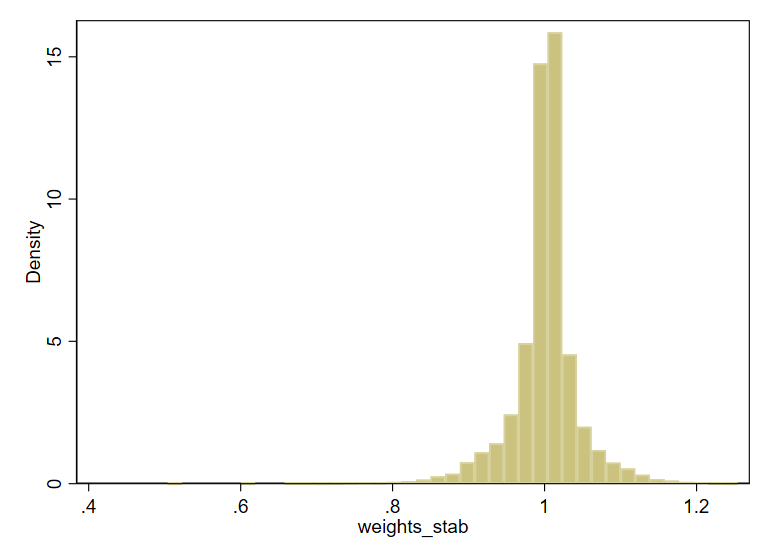

Supplement: Supplementary file 1 — Supplementary file1 (DOCX 184 KB) [file 15010_2026_2740_MOESM1_ESM.docx]
